# IL-17A Modulates Peritoneal Macrophage Recruitment and M2 Polarization in Endometriosis

**DOI:** 10.3389/fimmu.2020.00108

**Published:** 2020-02-14

**Authors:** Jessica E. Miller, Soo Hyun Ahn, Ryan M. Marks, Stephany P. Monsanto, Asgerally T. Fazleabas, Madhuri Koti, Chandrakant Tayade

**Affiliations:** ^1^Department of Biomedical and Molecular Sciences, Queen's University, Kingston, ON, Canada; ^2^Department of Obstetrics, Gynecology, and Reproductive Biology, Michigan State University, Grand Rapids, MI, United States

**Keywords:** M2 macrophage, interleukin-17A, endometriosis, inflammation, cytokines

## Abstract

Endometriosis is a debilitating gynecological disease characterized by the extrauterine presence of endometrial-like tissues located on the peritoneal membrane and organs of the pelvic cavity. Notably, dysfunctional immune activation in women with endometriosis could also contribute to the development of disease. In particular, alternatively activated (M2) peritoneal macrophages are shown to aid peritoneal lesion development by promoting remodeling of extracellular matrix and neovascularization of lesions. However, the stimuli responsible for polarizing M2 macrophages in endometriosis remain elusive. Interleukin-17A (IL-17A) can induce M2 macrophage polarization in other disease models and IL-17A is elevated in the plasma and endometriotic lesions of women with endometriosis. In this study, we investigated whether IL-17A could induce macrophage recruitment and M2 polarization, while promoting endometriotic lesion growth through enhanced vascularization. By utilizing a co-culture of macrophage-like THP-1 cells with an endometriotic epithelial cell line, our *in vitro* results suggest that IL-17A indirectly induces M2 markers CCL17 and CD206 by interacting with endometriotic epithelial cells. Further, in a syngeneic mouse model of endometriosis, IL-17A treatment increased macrophages in the peritoneum, which were also M2 in phenotype. However, IL-17A treatment did not augment proliferation or vascularization of the lesion in the study time frame. These findings suggest that IL-17A may be a stimulus inducing the pathogenic polarization of macrophages into the M2 phenotype by first acting on the endometriotic lesion itself.

## Introduction

Endometriosis is a gynecological disease characterized by the growth of endometrial-like lesions in extrauterine locations, including the peritoneum, ovaries and rectovaginal pouch (1). The cause of endometriosis is thought to derive from retrograde menstruation, where shed endometrial fragments travel via the fallopian tubes into the peritoneal cavity. Subsequent interaction with peritoneal structures, combined with evasion of pelvic immunity, allows these endometrial fragments to attach, invade, establish vascularization, and develop into endometriotic foci (2, 3). The mechanism of attachment, growth and disease severity has been difficult to elucidate due to the complex nature of the disease; however, it is now well-established that endometriosis is a chronic inflammatory condition and that the process of endometriotic lesion development is analogous to the process of wound healing.

Macrophages play a pathological role in the progression of endometriosis and its associated symptoms (4). Peritoneal macrophages from women with endometriosis are different as they produced increased amounts of vascular endothelial growth factor (VEGF) compared to peritoneal macrophages from healthy controls (5). An enrichment analysis of peritoneal cytokine and chemokine transcripts revealed that macrophage-produced cytokines were among the primary contributors of inflammation in women with endometriosis (6). Furthermore, in a mouse model of endometriosis, depletion of macrophages resulted in lesions that were reduced in size and vascularity (7), suggesting that macrophages are critical for the development of endometriotic lesions. A separate study further showed that the depletion of macrophages in an endometriosis mouse model drastically reduced markers of inflammatory pain and pain sensitivity behaviors (8).

Macrophages become activated via a classical (M1) or an alternative (M2) pathway, and recent studies have revealed that alternatively activated macrophages may be involved in the development of endometriosis. While M1 macrophages, due to its pro-inflammatory cytokine profile, promote tissue damage and worsen inflammatory disease progression, M2 macrophages are thought to participate in reducing inflammation. However, M2 macrophages can be pathogenic as they participate in extracellular matrix (ECM) remodeling on tissues wounded by acute and chronic inflammatory stimuli (9). Unfortunately, the polarization status of peritoneal macrophages in human patients remains debated in the literature, as both classically activated (M1) and alternatively activated (M2) phenotypes have been identified in the peritoneal fluid from women with endometriosis (6). This discrepancy could be explained by the heterogeneity of the disease and that perhaps different phenotypes of macrophages could be present at different times and stages of the disease. Indeed, processes such as ECM reconstruction and vascularization, which are associated with the progression of endometriotic lesions, are mediated by M2 macrophages (10–12). In many mouse models of endometriosis, it has been repeatedly shown that macrophages in the endometriotic lesion, as well as peritoneal macrophages, display an M2 polarized phenotype (7, 13, 14) and that M2 macrophages progress the disease by stimulating fibrogenesis and hyperalgesia (15). Specifically, bone marrow derived peritoneal macrophages, called small peritoneal macrophages (SPM), were shown to be increased and M2 polarized in an endometriosis mouse model, while resident macrophages called large peritoneal macrophages (LPM), had an M1 phenotype and were decreased (14). These changes in macrophage populations were accompanied by a rise in T helper 17 (T_H_17) cells and T regulatory cells (14). However, the stimuli that might be inducing these changes in macrophage population in endometriosis remains unknown.

Interleukin (IL)-17A is the main effector cytokine produced by T_H_17 cells. This proinflammatory cytokine has been shown to be involved in autoimmune diseases, mechanical injury, infection, cancer, obesity, and chronic inflammatory disorders (16). In the context of endometriosis, we and others have shown that IL-17A is elevated in the plasma and peritoneal fluid of women with endometriosis compared to controls and that endometriotic lesions produce IL-17 (17, 18). T_H_17 cells are also elevated in women with endometriosis compared to controls and women in advanced stages of endometriosis have higher numbers of T_H_17 cells compared to early stage patients with minimal/mild endometriosis (19–21). IL-17A is chemotactic for macrophages via its receptor, IL-17RA, and can also induce M2 polarization (22). In a mouse model of DSS-induced colitis, IL-17A was shown to exert its function by inducing development of M2-like macrophages in the lamina propria of the colon (23). Therefore, IL-17A could be the stimuli that mediates macrophage recruitment and M2 polarization in endometriosis.

Previously, we showed that treating endometrial cell lines with recombinant IL-17A can dose-dependently upregulate the production of pro-inflammatory cytokines and chemokines that recruit and activate macrophages, including G-CSF, GM-CSF, IL-8, CX3CL1 (17). Furthermore, treatment of the monocytic cell line, THP-1, with heterodimer IL-17A/F can increase the transcript levels of *IL-8, CXCL-1, TNF-*α, and *IL-23* (24), which are cytokines that have been found to be increased in the plasma of women with endometriosis (25). Based on these evidences, we hypothesized that IL-17A was a key signal involved in the recruitment of non-inflammatory and inflammatory monocytes, which are SPM precursors, to the peritoneal cavity and that it could induce M2 polarization of peritoneal SPMs. In this study, using both *in vitro* and *in vivo* approaches, we demonstrate previously unreported roles of IL-17A in macrophage recruitment and polarization in the context of endometriosis.

## Materials and Methods

### Human Cell Lines

THP-1 cells (TIB-202^TM^, American Type Culture Collection^©^, Manassas, VA), a monocytic cell line, and 12Z cells derived from epithelial cells of peritoneal endometriosis (provided by Anna Starzinski-Powitz) were incubated in a standard cell incubator at 37°C with 5% CO_2_. THP-1 cells were maintained in RPMI-1640 supplemented with 10% fetal bovine serum (FBS; Sigma-Aldrich, St. Louis, MO, USA). 12Z cells were maintained in DMEM/F12 (11330, Thermo Fisher Scientific, MA, USA) supplemented with 10% FBS (16000, Thermo Fisher Scientific, MA, USA), 1% penicillin and streptomycin (15140, Thermo Fisher Scientific, MA, USA) and 1% sodium pyruvate (11360, Thermo Fisher Scientific, MA, USA). Both cell lines were maintained in T75 cell culture flasks (Corning Inc., NY, USA) up to 70–80% confluence prior to experimental use.

### THP-1 Cells Differentiation and Treatment With Recombinant Human IL-17A

THP-1 cells were differentiated into macrophages using 10 ng/mL phorbol 12-myristate 13-acetate (PMA; Sigma Aldrich, St. Louis, MO, USA). Briefly, cells were incubated with 10 ng/mL PMA in 6 well plates (500,000–800,000 per well) and constituted in RPMI-1640 with 10% FBS for 48 h in a cell incubator at 37°C with 5% CO_2_. On day 3, media was replaced with PMA-free RPMI-1640 + 10% FBS, and then incubated for another 48 h. On day 6, PMA-differentiated THP-1 cells were treated with either 50 ng/mL recombinant hIL-17A (317-ILB-050, R&D systems, MN, USA) or phosphate buffered saline (PBS; Thermo Fisher Scientific, MA, USA). After 24 h, supernatant and cell pellets were collected and stored separately at −80°C until required for downstream applications. Supernatant samples collected from THP-1 cells treated with PMA and IL-17A (50 ng/mL) were subjected to human cytokine/chemokine 42-plex cytokine analysis (HD42; Eve Technologies Inc., Calgary, Alberta, Canada). The experiment was conducted in triplicate per treatment. Briefly, 1 ml of supernatant was collected and 50 μl were aliquoted and frozen in −80°C until sent for analysis to Eve Technologies, Inc. (Calgary, AB, Canada). The list of cytokines is as follows: EGF, Eotaxin-1, FGF-2, Flt-3L, CX3CL1, G-CSF, GM-CSF, GRO-α, IFN-α2, IFN-γ, IL-1α, IL-1β, IL-1ra, IL-2, IL-3, IL-4, IL-5, IL-6, IL-7, IL-8, IL-9, IL-10, IL-12(p40), IL-12(p70), IL-13, IL-15, IL-17A, IL-18, IP-10, MCP-1, MCP-3, MDC, MIP-1α, MIP-1β, PDGF-AA, PDGF-AB/BB, RANTES, sCD40L, TGF-α, TNF- β, VEGF-A.

### Treatment of 12Z Cells With Human IL-17A

12Z cells, which express IL-17RA, were treated with increasing concentrations of recombinant hIL-17A (5 ng/mL, 50 ng/mL, and 100 ng/mL) or PBS in DMEM/F12 (11330, Thermo Fisher Scientific, MA, USA) supplemented with 10% FBS (16000, Thermo Fisher Scientific, MA, USA), 1% penicillin and streptomycin (15140, Thermo Fisher Scientific, MA, USA), and 1% sodium pyruvate (11360, Thermo Fisher Scientific, MA, USA). After 24 h, supernatants were collected and sent to Eve Technologies Inc. (Calgary, AB, Canada) for multiplex cytokine analysis using the same human cytokine panel as described above.

### Treatment of THP-1 Cells With 12Z Conditioned Media

Triplicates of 12Z cells were cultured at 1 × 10^6^ cells/mL in 6 well plates and stimulated with either PBS or 50 ng/mL recombinant hIL-17A. After 24 h incubation in a standard cell culture incubator, the PBS or hIL-17-conditioned media (CM) from 12Z cells was transferred to 1 × 10^6^ cells/mL THP-1 cells in 6 well plates. The 12Z-CM was cultured with THP-1 cells for 24 h at 37°C with 5% CO_2._ After 24 h, THP-1 cells were pelleted and stored in −80°C until required for total RNA extraction.

### RNA Extraction, cDNA Synthesis, and Quantitative RT-PCR of THP-1 Cells

Total RNA was extracted from THP-1 cells, PMA-differentiated THP-1, and THP-1 cells treated with 12Z-CM using Norgen Biotek Total RNA isolation kit (17200, Norgen Biotek Corp. Thorold, ON, Canada) following manufacturer's instructions. PMA-differentiated THP-1 cells were trypsinized using 1X Trypsin-EDTA (Sigma Aldrich, St. Louis, MO. USA) and pelleted prior to total RNA extraction. For RNA extraction, cell pellets were incubated in 350 μL of lysis buffer solution and centrifuged at 15,000 rpm for 1 min. The resultant supernatant was then mixed with 250 μL of 100% ethanol and passed through a pre-assembled column to collect total RNA. RNA was reverse transcribed into complementary DNA (cDNA) using RT^2^ first strand kit (330404, Qiagen, USA) as per the manufacturer's protocol. The quantity and purity of RNA and cDNA were assessed using a Nanodrop 2000 Spectrophotometer (Thermo Scientific, MA, USA). Quantitative RT-PCR was conducted using LightCycler® 480 Real-Time PCR system (Roche Molecular Systems, Inc. Basel, Switzerland) with QuantiTect SYBR® Green PCR mastermix (Qiagen, Hilden, Germany). Relative gene expression values were calculated by the Roche LightCycler® 480 software using the threshold cycle number and normalized to the house keeping gene (ACTB) expression. The markers tested are as follows: M1 (*CSF3, PTGS2, IL1B, IL6, IL23, IL12, ccl5, ccl7, IFNG)* and M2 (*CD206, CD163, IL10, ccl17, PPARG*). The list of primers can be found in Table 1.

**Table 1:** Sequence of primers used for qRT-PCR.

**Primer**	**Sequence**
VEGF FOR	5′-GCCTTGCCTTGCTGCTCTA-3′
VEGF REV	5′-CACAGGATGGCTTGAAGATGTA-3′
TGF B FOR	5′-AAGGCTACGCCGCCTACTAC-3
TGFB REV	5′-CGGACCACCATGTTTCTGTAT-3′
IFN GAMMA FOR	5′-TGAGGGAACCAAACCAGAGA-3′
IFN GAMMA REV	5′-TCC TCT GGCTGCTGGTATTTA−3′
IL10 FOR	5′-AGCTGCTGCCTTGATTGTATT-3′
IL10 REV	5′-CGTGTGGGTTCAGCCTAGAT-3′
IL23 FOR	5′-TGAGGGAACCAAACCAGAGA-3′
IL23 REV	5′-CAGCAACAGCAGCATTACAG-3′
COX2 FOR	5′-ATGTTCCACCCGCAGTACA-3′
COX2 REV	5′-TTCTACCAGAAGGGCAGGATAC-3′
IL6 FOR	5′-CCAGAGCTGTCCAGATGAGTA−3′
IL6 REV	5′-TGACCTGCCCATGCTACA−3′
ACTB FOR	5′-CTGGACTTCGAGCAAGAGAT-3′
ACTB REV	5′-GATGTCCACGTCACACTTCA-3′
TNF FOR	5′-CTGCTGCACTTTGGAGTGAT-3′
TNF REV	5′-TGAAGAGGACCTGGGAGTAGA-3′
GMCSF FOR	5′-TTCCTGTGCAACCCAGATTA-3′
GMCSF REV	5′-ATTCTTCTGCCATGCCTGTATC-3′
CCL5 FOR	5′-GTGCCCACATCAAGGAGTATT-3′

### Generation of Bone Marrow Derived Macrophages

Bone-marrow derived macrophages (BMDMs) were generated from the long bones of female C57BL/6 mice per standard protocols (26). Briefly, mice were euthanized by CO2 narcosis and cervical dislocation and both femurs and tibias were excised and cleared of associated soft tissues. The distal and proximal epiphyses were cut with sterile scissors and the bone marrow was flushed with 5 mL of ice-cold calcium magnesium free PBS. Isolated bone marrow was manually disrupted using 20-gauge needle aspiration and filtered through 70 μm nylon mesh (10199-656; VWR, PA, USA) to prepared single-cell suspensions. Red blood cells were subsequently lysed using ACK solution (A10492-01; Thermo Fisher Scientific, MA, USA) as per the manufacturer's protocol. Haematopoietic precursors were resuspend in RPMI-1640 + 10% FBS (97068-085; VWR, PA, USA), 1x non-essential amino acids (11140050; Thermo Fisher Scientific, MA, USA), 100 U penicillin/100 μg/mL streptomycin (15140122; Thermo Fisher Scientific, MA, USA), seeded into 6-well plates and cultured for 7 days in the presence of 20 ng/mL recombinant mouse macrophage-colony stimulating factor (M-CSF; 576402 Biolegend). Purity of BMDMs was routinely assessed by flow cytometry and determine to be ≥97% CD11b + F4/80 + and >96% viable.

### Treatment of Bone Marrow Derived Macrophages With Recombinant Mouse IL-17 and Cytokine Analysis of the Supernatant

BMDMs were differentiated for 7 days in the presence of 20 ng/mL of recombinant mouse M-CSF as previously described. Cells were washed once with PBS and treated with either PBS or 5 ng/mL, 50 ng/mL, or 100 ng/mL of recombinant mouse IL-17 (7956-ML-100, R&D systems, MN, USA) (*n* = 6) for 24 h in the absence of M-CSF. Supernatants were collected, aliquoted and frozen in −80°C until sent for multiplex cytokine analysis to Eve Technologies, Inc. (MD31; Calgary, AB, Canada). Cytokines assessed include: Eotaxin, G-CSF, GM-CSF, IFN-γ, IL-1α, IL-1β, IL-2, IL-3, IL-4, IL-5, IL-6, IL-7, IL-9, IL-10, IL-12(p40), IL-12(p70), IL-13, IL-15, IL-17A, IP-10, KC, LIF, LIX, MCP-1, M-CSF, MIG, MIP-1α, MIP-1β, MIP-2, RANTES, TNF-α, and VEGF. Cells were harvested for flow cytometric staining using Versene solution (15040-066, Thermo Fisher Scientific, MA, USA).

### Flow Cytometric Analysis of Bone Marrow Derived Macrophages Treated With IL-17

BMDMs were harvested from the 6 well plate using Versene solution and immediately washed with PBS containing 2% FBS prior to immunostaining. Cells suspensions were stained with TruStain FcX (101319, Biolegend) and fixable viability dye eFluor 780 (65-0865-14; Thermo Fisher Scientific, MA, USA). The following surface marker antibodies and appropriate isotypes, all purchased from Biolegend unless otherwise specified, were used: Pacific Blue-anti-CD11b (M1/70), FITC-anti-F4/80 (BM8, Invitrogen), Per-CP-Cy5-5anti- I-A/I-E (M5/114.15.2), PE-Cy7-anti-CD115 (AFS98), and APC-anti-CD206 (FVS660, eBioscience). Cells were fixed and permeabilized using eBioscience intracellular fixation and permeabilization buffer set (88-8824-00; Thermo Fisher Scientific, MA, USA) following manufacturer's instructions. Following permeabilization, cells were stained with anti-CD206 (FVS660, eBioscience) for intracellular antigen detection. Cells were washed with PBS containing 2% FBS. Data was acquired using CytoFLEX S (Beckman Coulter, USA) and analyzed using Flowjo software.

### Surgical Induction of Endometriosis in C57BL/6 Female Mice

Eight to ten week old female C57BL/6 mice were purchased from Charles River Laboratories (USA) and housed in conventional cages with an automatic watering system and 12 h light/dark cycle at 3–4 animals per cage. To induce endometriosis, 3 mm fragments of uterus from donor female C57BL/6 mice were dissected, and 2 uterine fragments were surgically engrafted into each recipient female mouse. Briefly, each mouse was anesthetized using 4% isofluorane chamber. A small incision was made on the abdominal wall to gain access into the peritoneum. Two pieces of uterine fragments (~3 mm^2^/ fragment) were surgically adhered onto the peritoneum using Vetbond tissue adhesive (1469SB, 3M, MN, USA) followed by suturing of the peritoneum and stapling of the skin. All mice were provided with tramadol (30 mg/kg), bupivacaine (2 mg/kg), and saline via subcutaneous injection for 3 days following surgery to treat surgery induced pain and discomfort. Seven days after surgery, staples were removed, and blood samples were collected via submandibular puncture. Because endometriosis patients exhibit elevated levels of IL-17A systemically and in the endometriotic microenvironment, intraperitoneal injection of recombinant mouse IL-17A at 0.5 μg/mouse (7956-ML-100R&D systems, MN, USA,) began every 12 h for 7 days (*n* = 9). Intraperitoneal injection of sterile PBS with the same dosing schedule was used as a control (*n* = 9). After 7 days of treatment and before sacrifice, blood was collected via submandibular puncture. All animal studies were approved by Queen's University Animal Care Committee (Queen's University, Kingston, ON, Canada).

### Multiplex Cytokine Analysis (Mouse) on Plasma and Peritoneal Lavage From PBS and IL-17 Treated Mice

Whole blood obtained control and experimental mice was collected in K_2_EDTA-coated tubes (365974, BD Sciences, ON Canada). The blood was centrifuged for 15 min at 900 g and 4°C, plasma was then isolated, aliquoted and stored at −80°C. Using a mouse multiplex cytokine and chemokine array (MD31; Eve Technologies, Calgary, AB, Canada), cytokines were analyzed using the same mouse panel described above. Peritoneal lavage fluid (PF) was collected at the time of sacrifice via peritoneal lavage with 5 mL of ice-cold PBS and immediately put on ice. PF was centrifuged for 5 min at 400 g and 4°C, then PF was aliquoted and stored at −80°C. Cytokines were assessed with the same mouse multiplex cytokine and chemokine array as described above.

### Flow Cytometric Analysis of Peritoneal Fluid Cells

Following centrifugation of mouse PF samples and aliquoting of PF supernatant, remaining pelleted peritoneal cells were washed with PBS containing 2% FBS prior to immunostaining. Cells suspensions were first stained with TruStain FcX (101319; Biolegend, CA, USA) and fixable viability dye eFluor 780 (65-0865-14; Thermo Fisher Scientific, MA, USA). The following surface marker antibodies and appropriate isotypes, all purchased from Biolegend unless otherwise specified, were used: Alexa Fluor 700-anti-CD11b (M1/70), PE-anti-Ly6c (HK1.4), FITC-anti-F4/80 (BM8, Invitrogen), Per-CP-Cy5-5anti- I-A/I-E (M5/114.15.2), PE-Cy7-anti-CD115 (AFS98), and APC-anti-CD206 (FVS660, eBioscience). Cells were fixed and permeabilized using eBioscience intracellular fixation and permeabilization buffer set (88-8824-00; Thermo Fisher Scientific, MA, USA) following manufacturer's instructions. Following permeabilization, cells were stained with anti-CD206 (FVS660, eBioscience) for intracellular antigen detection. Cells were washed with FACS buffer. Data was acquired using FACS ARIA III (BD Biosciences, USA) and analyzed using Flowjo software. Non-inflammatory monocytes were gated as live, CD11b+Ly6c+, MHCII- cells, inflammatory monocytes as live, CDllb+, Ly6c+, MHCII+ cells, SPM as live, CD11b+ CD115+, F4/80 intermediate, MHCII+ cells, and LPM as live, CD11b+ CD115+, F4/80+, MHCII- cells. CD206, which is a well-established M2 marker, was used to characterize the M2 status (**Figure 5K**).

### Immunolocalization of CD31^+^ Endothelium, and Ki67^+^ Proliferation in Mouse Endometriosis-Like Lesions

Endometriosis-like lesions were excised and fixed in 4% paraformaldehyde for 16 h at 4°C, then transferred to 70% ethanol prior to paraffin embedding. Paraffin embedded lesions were cut at 5 μm in thickness. Antigen retrieval was conducted with cell condition 1 for 60 min and stained with rabbit anti-mouse ki67 (ab 15580, Abcam; 1:1000) and rabbit anti-mouse CD31 (77699S, New England Biolabs, 1:100) using Ventana Discovery immunostainer (Ventana Medical Systems, Inc., USA) at the Department of Pathology at Queen's University (Kingston, ON, Canada). Anti-rabbit secondary antibodies were stained for 60 min and Ultrablue DAB detection kit was used (Ventana Medical System Inc). Then, the slides were stained with haematoxylin and bluing reagent for 4 min for the counterstain, and a coverslip was applied. The slides were scanned using Aperio ScanScope SC slide scanner (Leica Biosystems Imaging, Inc., Germany), and images taken using Aperio ImageScope (Leica Biosystems Imaging, Inc., Germany). Semi-quantitative analysis of Ki67 and CD31 was conducted using ImageJ by setting the threshold of positive staining using three representative images, then calculating the percent positive staining per slide.

### Statistical Analysis

GraphPad Prism® 7.02 Software was used for statistical analysis. Unpaired *t*-test with Welch's correction was used when means of two independent groups were tested for statistical significance. Results with *P* ≤ 0.05 were considered statistically significant.

## Results

### PMA-Differentiated THP-1 Cells Treated With IL-17A Produced Macrophage Associated Cytokines and Showed Elevated mRNA of Macrophage Associated Markers

We previously showed that women with endometriosis have increased circulating levels of IL-17A compared to healthy fertile controls and that human endometriotic lesions produce IL-17 (17). Because macrophages play an important role in the progression of endometriosis, we wanted to assess the effect of IL-17A on monocytes and macrophages. To do this, we used an *in vitro* model to test whether recombinant hIL-17A could elicit the expression of macrophage associated cytokines and chemokines as well as M1 or M2 markers in both naïve THP-1 cells and THP-1 cells differentiated into macrophages using PMA.

**Figure 1:**
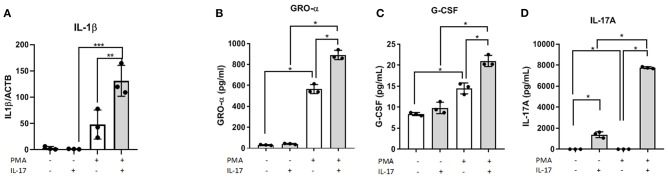
IL-17A induces *IL1B* expression and, GRO- α and G-CSF production in PMA-differentiated THP-1 cells. THP-1 cells were differentiated into macrophages using PMA (10 ng/mL) for 48 h followed by IL-17A (50 ng/mL) stimulation for 24 h in PMA-free media. Total RNA was collected from differentiated THP-1 cells treated with either without PMA and with PMA and treated with either PBS or IL-17A. Semi-quantitative RT-PCR revealed *IL1B* to be upregulated in IL-17A-treated cells **(A)**. In the supernatant collected following 24 h incubation with IL-17A, we detected increased concentration of GRO-α **(B)**, G-CSF **(C)** and IL-17A **(D)** in differentiated THP-1 cells (gray columns) compared to non-differentiated THP-1 cells (white columns). Statistical analysis conducted using One-way ANOVA with Bonferroni *post-hoc* test. *P ≤ 0.05, **P ≤ 0.01, ***P ≤ 0.001.

In the supernatant of differentiated THP-1 cells treated with 50 ng/mL of IL-17A, we observed significantly increased concentrations of macrophage associated cytokines including GRO-α (Figure 1B) and G-CSF (Figure 1C). Compared to undifferentiated THP-1 cells treated with IL-17A, differentiated THP-1 cells treated with IL-17A had elevated IL-17A in the supernatant (Figure 1D), which suggests that the differentiated THP-1 cells may undergo an autocrine-induced upregulation of IL-17A production compared to the undifferentiated THP-1 monocytes. This upregulation of IL-17A with PMA treatment was not due to PMA treatment, as we did not see IL-17A production in cells treated with PMA and PBS (PMA/PBS; Figure 1C). We also evaluated mRNA expression of known M1 and M2 macrophage markers and cytokines and chemokines in THP-1 cells that were differentiated into macrophages using PMA (10 ng/mL), then subjected to either IL-17A (50 ng/mL) or PBS treatment. From the markers tested for M1 (*CSF3, PTGS2, IL1B, IL6, IL23, IL12, ccl5, ccl7, IFNG)* and M2 (*CD206, CD163, IL10, ccl17, PPARG*), *IL1B* showed statistical significance in PMA-differentiated macrophages with IL-17A treatment (Figure 1A).

### Endometriotic Epithelial (12Z) Cells Treated With IL-17A Secrete Macrophage Associated Cytokines and Chemokines

**Figure 2:**
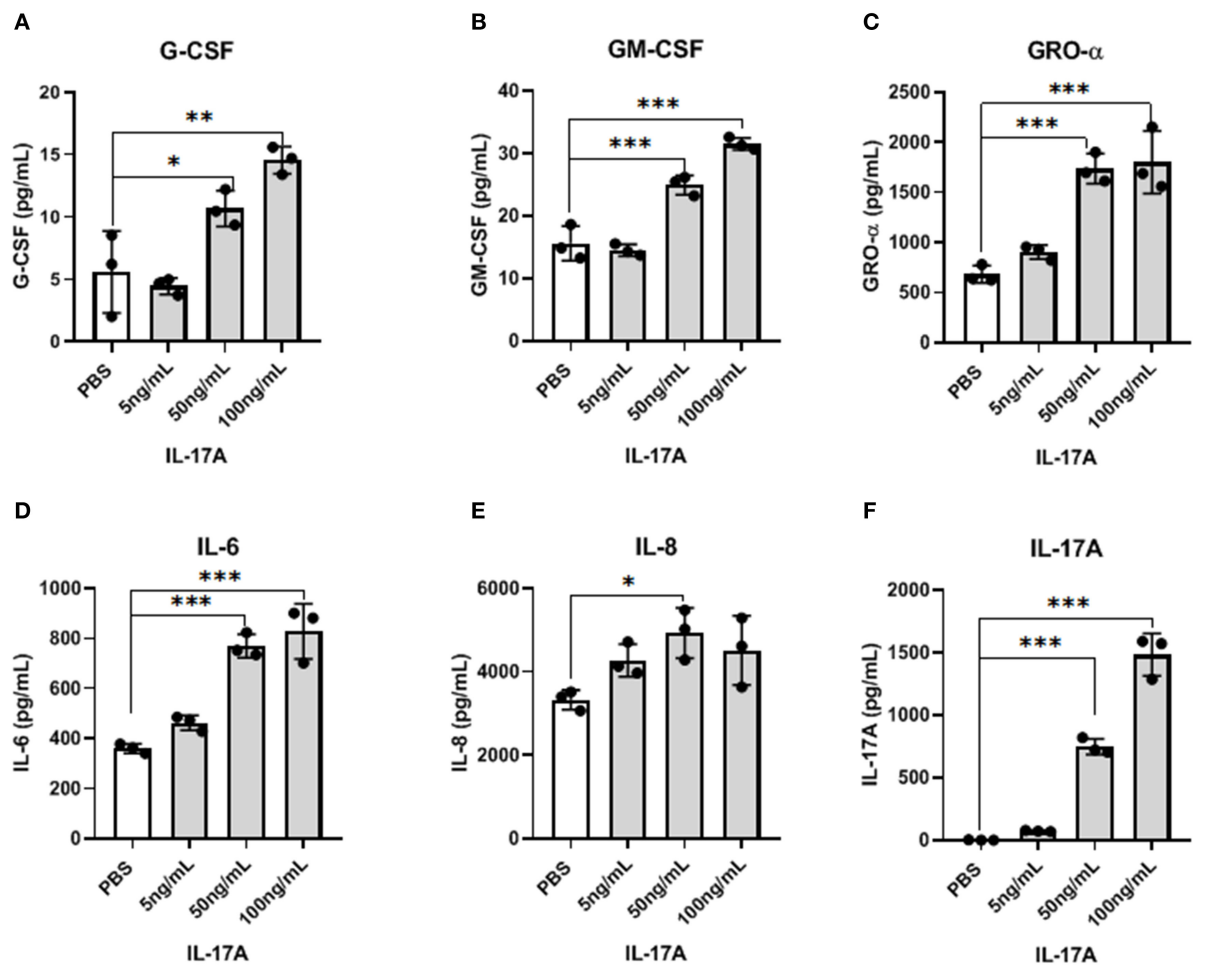
IL-17A induces the production of inflammatory cytokines and chemokines from 12Z cells in a dose-dependent manner. 12Z cells were plated in 6 wells at 1 × 10^6^/ well and incubated for 24 h with different concentration of IL-17A (5 ng/mL, 50 ng/mL, and 100 ng/mL), as well as with PBS as control. Out of 42 cytokines analyzed in the supernatant, G-CSF **(A)**, GM-CSF **(B)**, GRO-α **(C)**, IL-6 **(D)**, and IL-8 **(E)** showed dose-dependent increase in concentration that was significantly different from the level obtained with PBS treatment. Statistical analysis conducted using one-way ANOVA with Tukey *post-hoc* test **(A–F)**. *P ≤ 0.05, **P ≤ 0.01, ***P ≤ 0.001.

Because IL-17A acts on a multitude of cell types to elicit its functions, we hypothesized that IL-17A might be interacting with and signaling through the endometriotic lesion itself. To test this, 12Z cells, derived from epithelial cells of peritoneal endometriosis, were treated with hIL-17A to measure the cytokine and chemokine response. Out of 42 cytokines analyzed, macrophage associated cytokines and chemokines including G-CSF, GM-CSF, GRO-α, IL-6, and IL-8 were dose dependently increased in the supernatant of 12Z cells compared to PBS-treated controls (Figures 2A–F). This suggests that IL-17A could be upregulating macrophage modulating cytokines and chemokines and thereby modulating macrophages indirectly by first signaling through the epithelial cells in the lesion.

### THP-1 Cells Treated With Conditioned Media From IL-17A-Treated 12Z Cells Promotes Markers That Indicate an M2 Polarized Phenotype

**Figure 3:**
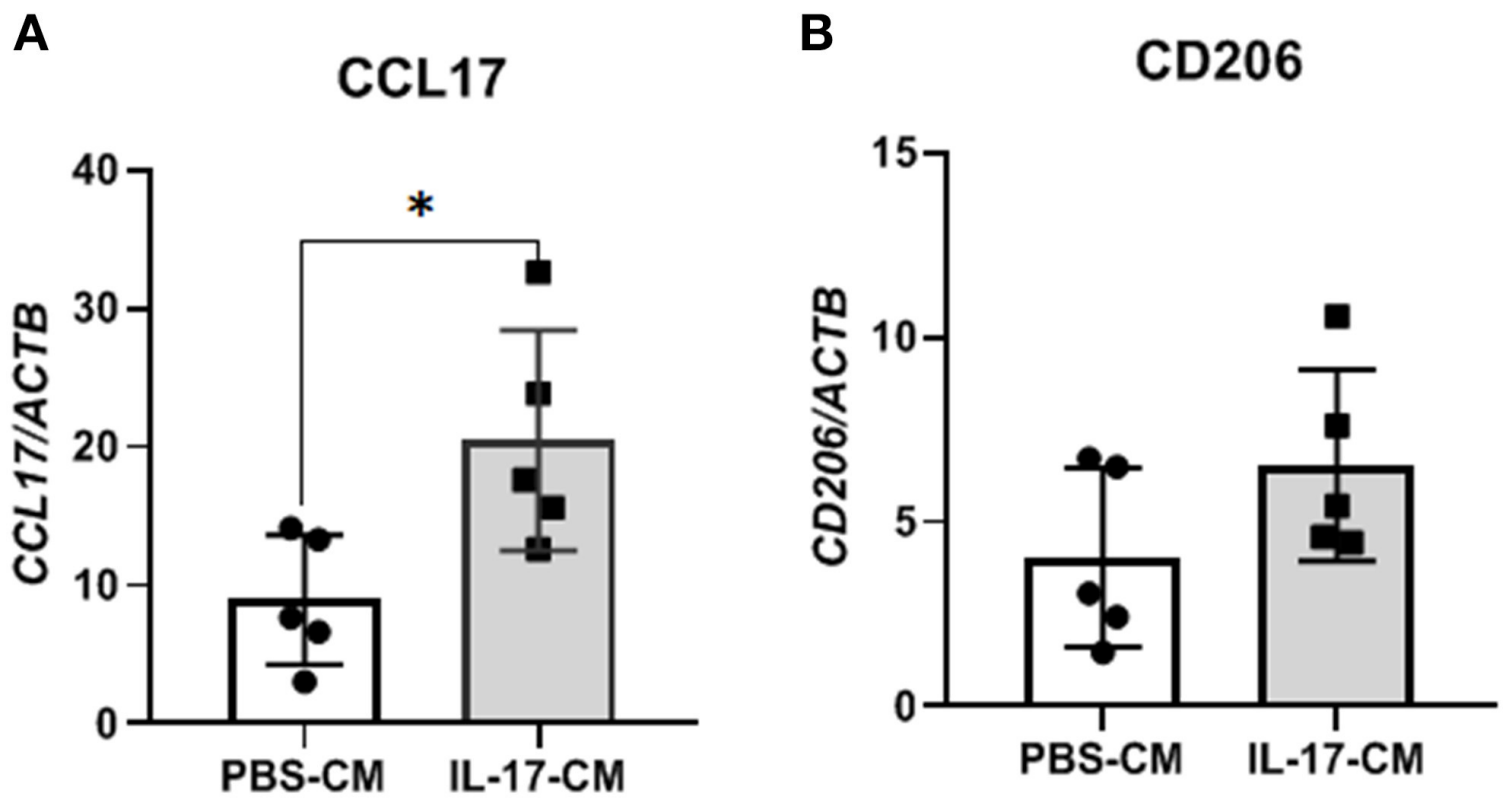
THP-1 cells incubated with conditioned media from 12Z cells treated with IL-17A showed alterations in M2 polarization markers. 12Z cells were plated at 1 x 10^6^ cells per well and incubated for 24 h with either PBS or 50 ng/mL of IL-17A. THP-1 cells were then incubated with this conditioned media for 24 h. Total RNA was extracted from THP-1 cells to test for M1 and M2 markers via RT-qPCR. Marker for M2 macrophages, *ccl17* **(A)** was significantly upregulated in THP-1 cells treated with IL-17A-CM compared to PBS-CM. Transcription level of *CD206* **(B)**, also a marker of M2 macrophage, was increased in IL-17A-CM treated cells; however, the difference did not reach statistical significance. Statistical analysis conducted using an unpaired *t*-test with Welch's correction **(A,B)**. *P ≤ 0.05.

To test whether indirect signaling of IL-17A through 12Z cells could be modulating macrophages and the M2 polarization of macrophages, we again treated 12Z cells with either PBS or IL-17A and put that resulting conditioned media (PBS-CM or IL-17A-CM) onto naïve THP-1 cells. This test was used to assess whether the products of 12Z cells, following PBS or IL-17A, stimulation would be able to polarize macrophages to an M2 phenotype from naïve THP-1 cells. After overnight incubation in either IL-17A-CM or PBS-CM, we collected the THP-1 cells and extracted total RNA to assess gene expression of markers of both M1 (*CSF3, PTGS2, IL1B, IL6, IL23, IL12, ccl5, ccl7, IFNG)* and M2 (*CD206, CD163, IL10, ccl17, PPARG*). We observed that IL-17A-CM induced significant upregulation of *ccl17*, a marker of M2 macrophages, in naïve THP-1 cells compared to PBS-CM (Figure 3A). Additionally, mRNA level of CD206 was also increased in cells treated with IL-17A-CM compared to PBS-CM; however, the difference did not reach statistical significance (Figure 3B).

### Bone Marrow Derived Macrophages (BMDM) Treated With Recombinant Mouse IL-17A Did Not Exhibit Altered M2 Polarized Phenotype but Released Macrophage Associated Cytokines

**Figure 4:**
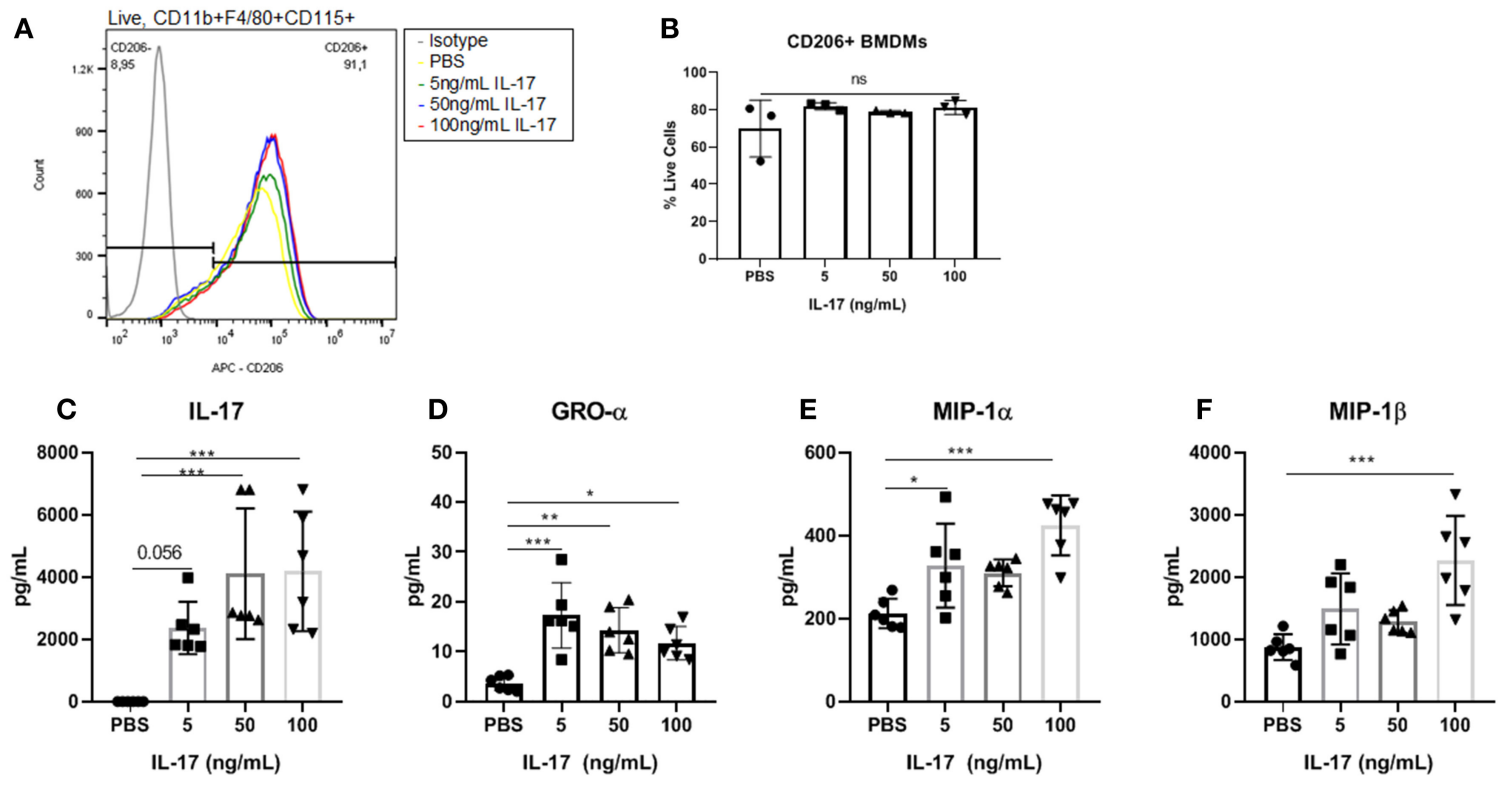
Bone marrow derived macrophages treated with IL-17 secreted elevated levels of macrophage associated cytokines however showed no differences in M2 polarization status compared to PBS-treated controls. **(A)** Histogram showing no difference in CD206 expression between BMDMs (Live, CD11b+, F4/80+, CD115+ cells) treated with PBS compared to treated with 5, 50, and 100 ng/mL of rmIL-17. **(B)** No significant differences were found in CD206+ BMDMs treated with IL-17 compared to PBS controls. Out of 31 analytes, IL-17 **(C)**, GRO-α **(D)**, MIP-1α **(E)**, and MIP-1β **(F)** were found to be significantly elevated in the supernatant of IL-17 treated BMDMs compared to PBS controls. Statistical analysis conducted using one-way ANOVA with Tukey *post-hoc* test **(B–F)**. *P ≤ 0.05, **P ≤ 0.01, ***P ≤ 0.001.

Similar to the results demonstrated with hrIL-17A treated THP-1 cells, the treatment of recombinant mouse IL-17 (rmIL-17) did not alter the M2 polarization status of mouse BMDM (Figures 4A,B). However, treatment with rmIL-17A dose dependently increased the production of macrophage associated cytokines including GRO-α, MIP-1α, and MIP-1β (Figures 4C–F).

### Small Peritoneal Macrophages Are Elevated in IL-17A-Treated Mice and Exhibit an M2 Polarized Phenotype

**Figure 5:**
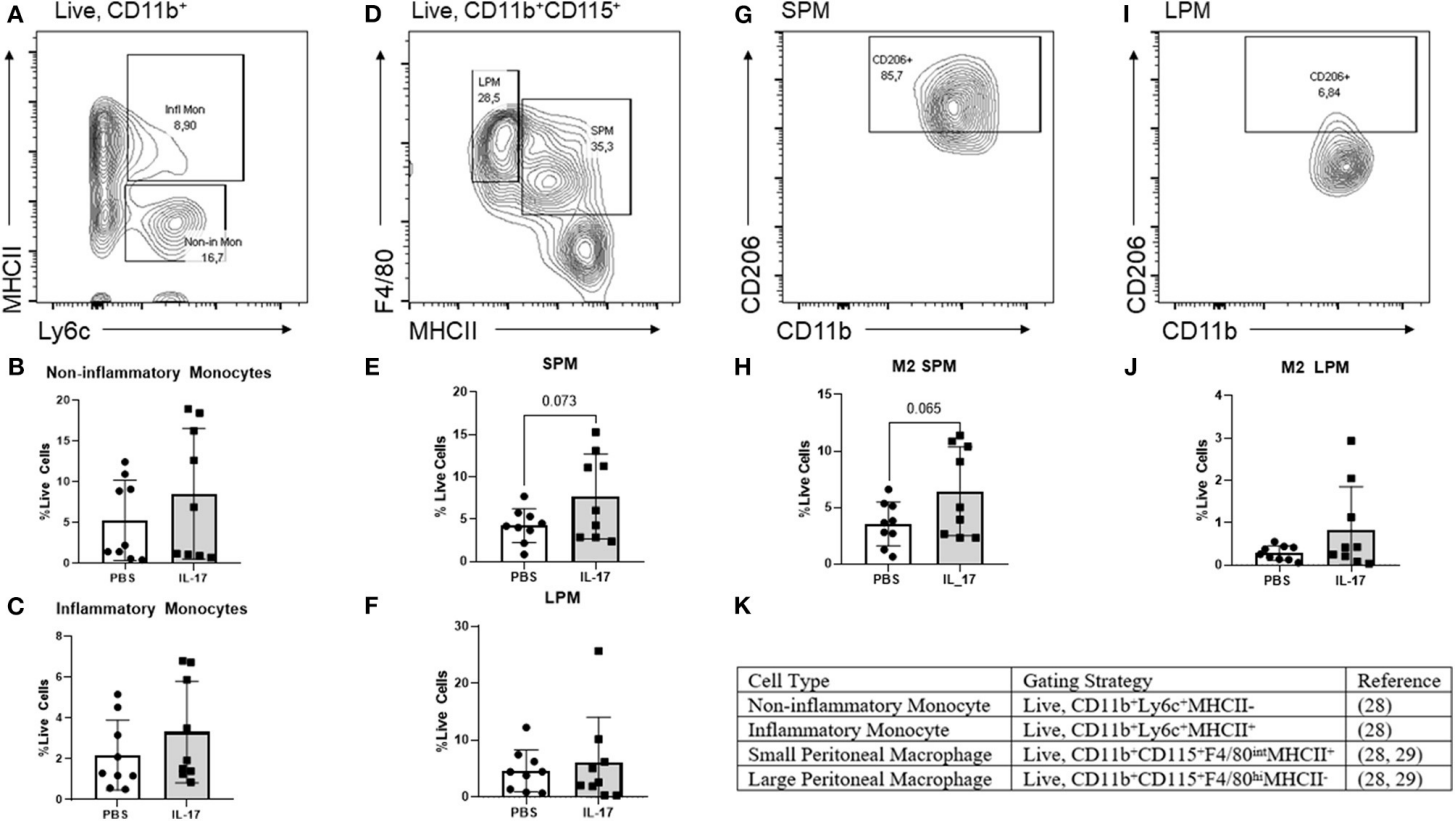
Characterization of monocytes and macrophages in the peritoneal lavage fluid of mice treated with IL-17A or PBS. **(A,D,G,I,K)** Gating strategy for monocytes, non-inflammatory monocytes, inflammatory monocytes, small peritoneal macrophage (SPM), large peritoneal macrophage (LPM) and CD206 polarization status. **(B,C)** Scatter plot for non-inflammatory and inflammatory monocytes shows no difference between IL-17A (*n* = 9) and PBS group (*n* = 9). **(E)** Scatter plot for % Live cells peritoneal lavage fluid shows elevated SPM in the IL-17A (*n* = 9) treated group compared to PBS group (*n* = 9). **(F)** No difference was found in the populations of LPM. **(H,J)** SPM had elevated CD206 in the IL-17A-treated group (*n* = 9) compared to the PBS-treated group (*n* = 9). No significant differences in CD206 were found in LPM. Scatter plot for % F4/80^+^CD206^+^ cells from CD11b^hi^ population show no difference between IL-17A (*n* = 9) and PBS group (*n* = 9) based on unpaired *t*-test with Welch's correction.

From our experiments *in vitro*, we postulated that IL-17A could be acting both on the lesion and on monocytes/macrophages to recruit and polarize macrophages to an M2 phenotype. To explore this further, we used a mouse model of endometriosis using C57BL/6 female mice and treated them with either rmIL-17A (0.5 ug/100 uL) or PBS (control). We investigated the effect of rmIL-17 treatment on monocyte populations, macrophage populations, and M2 polarization status in the peritoneal cavity of mice induced with endometriosis. No differences were found in populations of non-inflammatory monocytes in the peritoneal cavity (Figures 5A,B). IL-17A-treated mice had a trend of increased numbers of inflammatory monocytes in the peritoneal cavity (Figures 5A,C) although the difference did not reach statistical significance. However, IL-17A-treated mice had elevated levels of small peritoneal macrophages (SPM) (Figures 5D,E) as well as CD206+ SPM (Figures 5G,H) compared to PBS mice. Large peritoneal macrophages (LPM) populations were not altered (Figures 5D,F) and showed little CD206 expression (Figures 5I,J). Together this suggests that IL-17A treatment could be mobilizing monocytes from the bone marrow leading to increased SPM populations in the peritoneal cavity of IL-17A-treated mice.

### Plasma and PF Cytokine Profiles in Mice Induced With Endometriosis Does Not Differ Between rmIL-17A or PBS-Treated Mice

**Figure 6:**
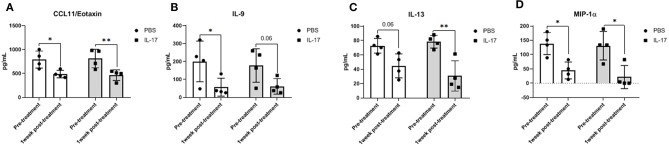
Multiplex cytokine analysis in plasma samples of mice treated with IL-17A (0.5 μg/100 μl). Out of 31 analytes assessed, Eotaxin **(A)**, IL-9 **(B)**, IL-13 **(C)**, and MIP-α **(D)** were decreased in plasma after 1 week of both PBS (*n* = 4) and IL-17A (*n* = 4) treatment. Repeated measurement 2-way ANOVA with Sidak multiple comparison correction method was used as statistical analysis. *P ≤ 0.05, **P ≤ 0.01.

**Figure 7:**
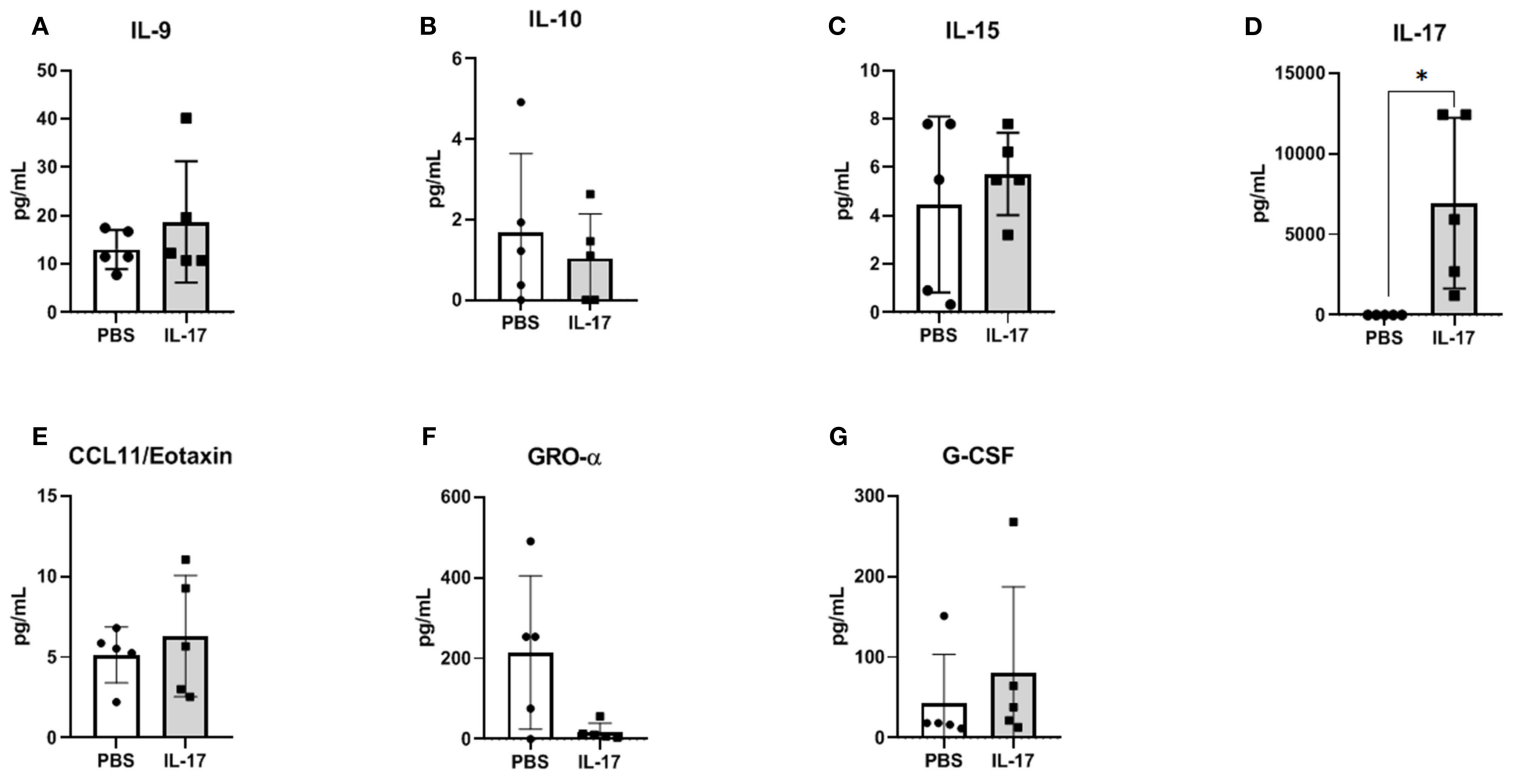
Multiplex cytokine analysis in peritoneal fluid of mice treated with IL-17A (0.5 μg/100 μl). Out of 31 analytes assessed, IL-9 **(A)**, IL-10 **(B)**, IL-15 **(C)**, IL-17A **(D)**, CCL11/Eotaxin **(E)**, CXCL1/KC **(F)**, and G-CSF **(G)** were detected; however, no significant differences were found between PBS (*n* = 4) and IL-17 (*n* = 4) treated mice. Undetected analytes are not shown. Unpaired *t*-test was used as statistical analysis. *P ≤ 0.05.

Compared to PBS controls, IL-17A treatment in mice lead to no significant alterations in inflammatory and macrophage-recruiting cytokine profile in the plasma or PF lavage. When plasma cytokine levels between pre-treatment and 1 week post-treatment were compared, mice treated with IL-17A (0.5 ug/100 uL) displayed decreased plasma levels of IL-13, IL-9, MIP-1α, and Eotaxin (Figures 6A–D, respectively**)**. However, PBS-Treated Mice also showed decreased plasma levels of Eotaxin, IL-9, IL-13, and MIP-1α (Figures 6A–D), suggesting that the difference may stem from the induction endometriosis, and not from the IL-17A injection. No significant differences were found in PF (Figures 7A–G).

### Recombinant IL-17A Treatment in Mice Induced With Endometriosis Does Not Alter Lesion Development, Vascularization, or Proliferation

**Figure 8:**
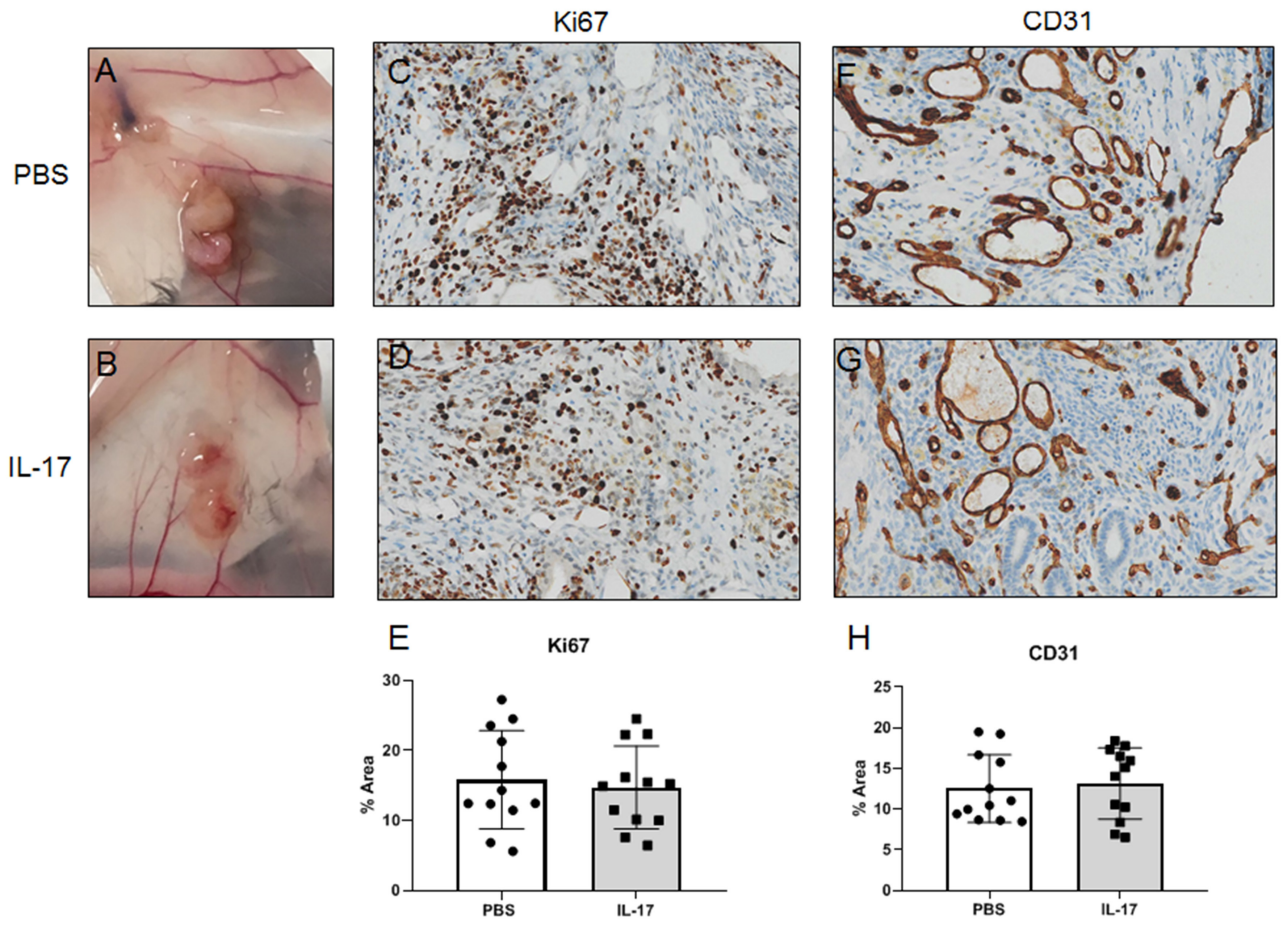
Gross and histological images of endometriosis lesions in mice treated with IL-17A or PBS. PBS (**A**; *n* = 4) and IL-17A (**B**, *n* = 4). Both PBS-treated **(C)** and IL-17A-treated **(D)** lesions had Ki67+ cells within the stromal region of the tissue, with occasional positivity in the luminal epithelium. Semi quantitative analysis revealed no significant differences in Ki67 staining. Both IL-17A-treated **(F)** and PBS control lesions **(G)** show extensive vascularization within the stroma as denoted by cross sections of vessels stained in brown. Semi-quantitative analysis showed no significant difference **(E,H)**. All images are taken at 200x magnification.

Gross evaluation of endometriosis lesions did not reveal any major macroscopic anomalies for size and appearance of lesions in mice treated with either IL-17A or PBS. Figures 8A,B represents prototypical appearance of endometriosis-like lesions established on mouse peritoneal membrane. As we and others have shown, IL-17A is a cytokine that induces pro-angiogenic (i.e. VEGF) and pro-inflammatory (i.e., IL-6, IL-8) cytokines from epithelial cells (17, 27). To establish whether IL-17A treatment would impact vascularization and growth of endometriotic lesions in mice, we used anti-Ki67 and anti-CD31 staining to assess proliferation (Figures 8C,D) and vascularization (Figures 8F,G). As demonstrated in Figure 8G, we observed robust CD31 staining in the lesions treated with IL-17. However, semi-quantitative analysis did not reveal significant differences between the treatment groups (Figures 8E,H). Additionally, Ki67+ cells were also comparable between IL-17A-treated and PBS control lesions (28, 29).

## Discussion

**Figure 9:**
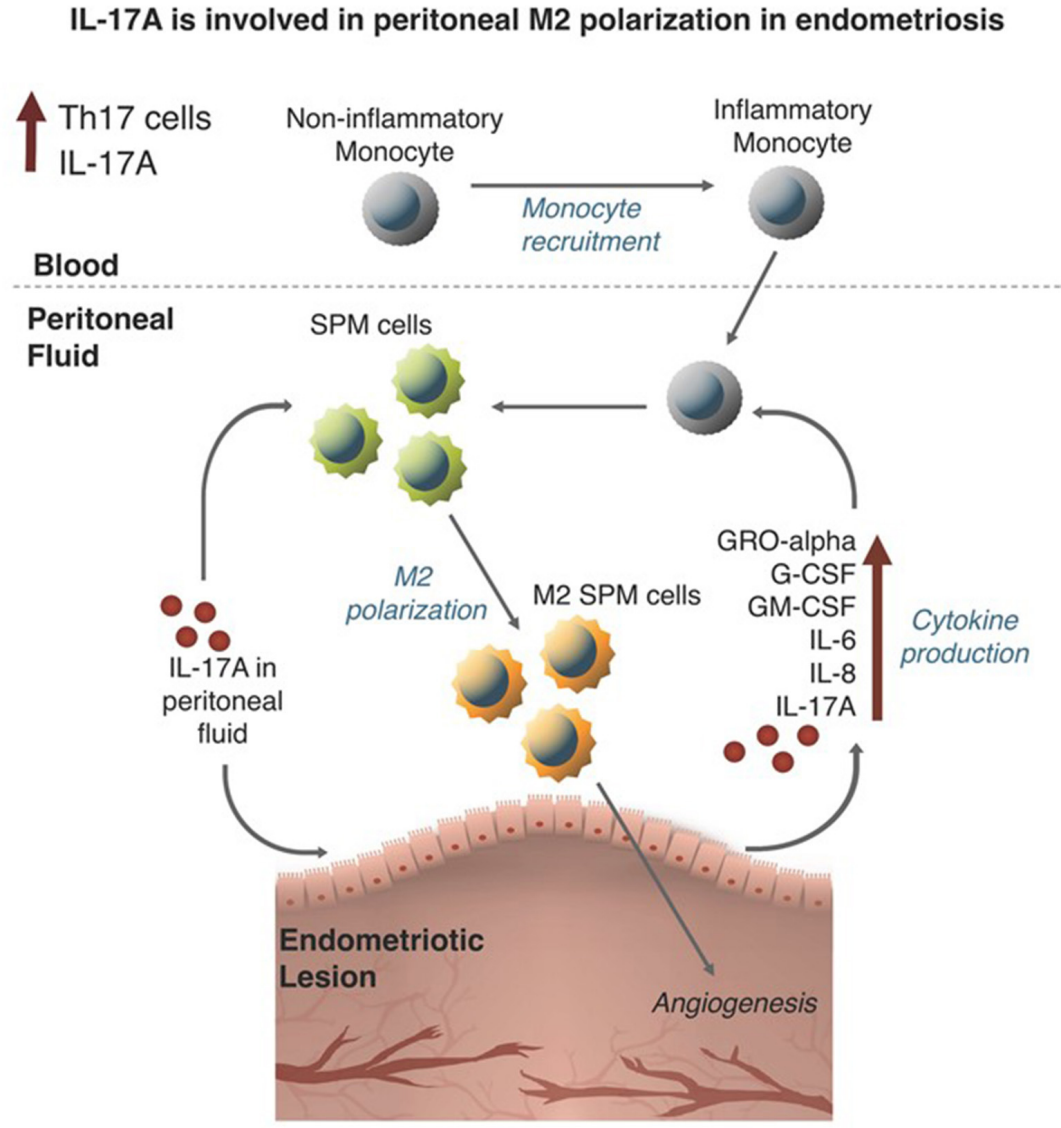
Visual Summary: IL-17A is involved in peritoneal M2 polarization in endometriosis.

The pathogenic role of macrophages has been well-documented in endometriosis. Many reports have focused on the role of M2 macrophages in the progression of the disease and development of symptoms. However, the specific stimulus leading to the recruitment and polarization of macrophages in endometriosis was unknown. In this study, we assessed the contribution of elevated IL-17A to macrophage recruitment and polarization in the endometriotic microenvironment using both an *in vitro* and *in vivo* experimental approach (summarized in Figure 9). The premise of this study was based on the evidence demonstrating elevated IL-17A levels, elevated numbers of Th17 cells, and the involvement of peritoneal macrophages in the development and vascularization of endometriotic lesions established in mice (7, 30). Furthermore, in other disease models, IL-17A participates in monocyte recruitment to sites of inflammation (31) and promotes M2 polarization in monocyte derived macrophages (32). Although studies have shown the potential role of IL-17A in the direct polarization of macrophages into M2 phenotype (33, 34), it was unknown whether IL-17A plays a similar role in endometriosis.

Our *in vitro* results showed the capacity of IL-17A to induce a heterogeneous response from THP-1 cells depending on the differentiation status of THP-1 cells as monocyte-like or macrophage-like. PMA-differentiated THP-1 cells (macrophage-like status) treated with IL-17A showed increased mRNA expression of *IL1B* and increased protein production of GRO-α and G-CSF in the supernatant compared to PBS-treated or monocyte-like THP-1 cells. Both GRO-α and G-CSF are known neutrophil-associated chemokines, which suggest that IL-17A acting directly on macrophages could be mediating neutrophil infiltration to the sites of inflammation via inducing the production of chemokines and growth factors like GRO-α and G-CSF (35–38). This finding prompts further investigation into the interplay between neutrophils and macrophages in endometriosis pathogenesis, which could potentially be mediated by IL-17A. Similarly, treating mouse BMDMs with rmIL-17A did not alter the expression of CD206 compared to PBS-treated macrophages. However, like THP-1 cells treated with IL-17A, the supernatant of IL-17A-treated BMDMs showed elevated levels of macrophage associated cytokines including GRO-α, MIP-1α, and MIP-1β. Therefore, IL-17A alone did not directly induce a M2 phenotypic change of macrophage-like THP-1 cells or BMDMs; however, IL-17A increased their expression of macrophage associated cytokines.

Interestingly, IL-17A treatment of 12Z cells, the endometriotic epithelial cell line, produced macrophage activating and recruiting cytokines including G-CSF, GM-CSF, GRO-α, and IL-8. This result suggests that IL-17A may induce changes in peritoneal macrophages by first interacting with the endometriotic lesion itself. Therefore, we incubated 12Z cells with IL-17A and used this supernatant to treat macrophage-like THP-1 cells. We found that transcripts for M2 markers including *CCL17* and *CD206* were increased. This finding indeed suggests that the products of 12Z cells following IL-17A stimulation could be contributing to the recruitment and M2 polarization of peritoneal macrophages in endometriosis. However, transcript levels of other M2 markers *IL10* and *IL12*, were undetectable in the same THP-1 cells treated with either IL-17A or PBS conditioned media from 12Z cells.

To further understand both the direct and indirect role of IL-17A in the recruitment and polarization of peritoneal macrophages into an M2 phenotype, we used our well-established mouse model of endometriosis. After 7 days of IL-17A treatment, we found no significant differences in inflammatory or non-inflammatory monocytes in the peritoneal environment; however, there was a detectable but non-significant increase in inflammatory monocytes in the peritoneal lavage fluid. We did find elevated SPM numbers in the IL-17A-treated mice compared to PBS controls (*P* = 0.07), which suggests that IL-17A treatment stimulated the recruitment of bone marrow derived SPM precursors and SPM. Additionally, the SPM were CD206+, suggesting that IL-17A is involved in the polarization into M2 phenotype. This finding supports previous reports showing that SPMs polarize to an M2 phenotype in a mouse model of endometriosis and that this shift coincides with elevated levels of Th17 cells (14). Therefore, together with our *in vitro* data, we suggest IL-17A is involved in macrophage recruitment and may be indirectly polarizing SPM into a pathogenic M2 phenotype by first interacting with the endometriotic lesion.

Despite previous *in vitro* evidence demonstrating the potential of IL-17A to promote angiogenesis by inducing the production of VEGF and PDGF (39), we did not observe alterations in vascularization or proliferation of lesions in mice treated with IL-17A compared to PBS. However, this could be due to the short duration of the experiment, as larger, observable changes in growth and vascularization could take longer than 7 days to develop.

This study provides a basis for future investigations to examine the response of macrophage populations to the elevated systemic levels of IL-17A in women with endometriosis. Using a single cell RNAseq approach from fresh samples of endometriotic lesions stratified by stages of the disease may allow identification of endometriosis-associated macrophages and distinguish their polarization state. Such foundational human data will propel our currently limited knowledge on the role of innate immune cells in endometriosis, especially peritoneal macrophages and T_H_17 cells and the way they interact with the local inflammatory peritoneal environment in endometriotic lesion development.

## Data Availability Statement

The datasets generated for this study are available on request to the corresponding author.

## Ethics Statement

The animal study was reviewed and approved by Queen's University Animal Care Committee.

## Author Contributions

JM and SA conceived experiments, conducted experiments, analyzed data, and wrote the manuscript. RM and SM conducted experiments. AF contributed reagents. MK and CT contributed reagents, conceived experiments, provided financial support, and editing of manuscript.

### Conflict of Interest

The authors declare that the research was conducted in the absence of any commercial or financial relationships that could be construed as a potential conflict of interest.
